# Characteristics of Health Care Personnel with COVID-19 — United States, February 12–April 9, 2020

**DOI:** 10.15585/mmwr.mm6915e6

**Published:** 2020-04-17

**Authors:** CDC COVID-19 Response Team, CDC COVID-19 Response Team, Sherry L. Burrer, Marie A. de Perio, Michelle M. Hughes, David T. Kuhar, Sara E. Luckhaupt, Clinton J. McDaniel, Rachael M. Porter, Benjamin Silk, Matthew J. Stuckey, Maroya Walters

**Affiliations:** CDC; CDC; CDC; CDC; CDC; CDC; CDC; CDC; CDC; CDC.

As of April 9, 2020, the coronavirus disease 2019 (COVID-19) pandemic had resulted in 1,521,252 cases and 92,798 deaths worldwide, including 459,165 cases and 16,570 deaths in the United States (*1*,*2*). Health care personnel (HCP) are essential workers defined as paid and unpaid persons serving in health care settings who have the potential for direct or indirect exposure to patients or infectious materials (*3*). During February 12–April 9, among 315,531 COVID-19 cases reported to CDC using a standardized form, 49,370 (16%) included data on whether the patient was a health care worker in the United States; including 9,282 (19%) who were identified as HCP. Among HCP patients with data available, the median age was 42 years (interquartile range [IQR] = 32–54 years), 6,603 (73%) were female, and 1,779 (38%) reported at least one underlying health condition. Among HCP patients with data on health care, household, and community exposures, 780 (55%) reported contact with a COVID-19 patient only in health care settings. Although 4,336 (92%) HCP patients reported having at least one symptom among fever, cough, or shortness of breath, the remaining 8% did not report any of these symptoms. Most HCP with COVID-19 (6,760, 90%) were not hospitalized; however, severe outcomes, including 27 deaths, occurred across all age groups; deaths most frequently occurred in HCP aged ≥65 years. These preliminary findings highlight that whether HCP acquire infection at work or in the community, it is necessary to protect the health and safety of this essential national workforce.

Data from laboratory-confirmed COVID-19 cases voluntarily reported to CDC from 50 states, four U.S. territories and affiliated islands, and the District of Columbia, during February 12–April 9 were analyzed. Cases among persons repatriated to the United States from Wuhan, China, and the Diamond Princess cruise ship during January and February were excluded. Public health departments report COVID-19 cases to CDC using a standardized case report form* that collects information on patient demographics, whether the patient is a U.S. health care worker, symptom onset date, specimen collection dates, history of exposures in the 14 days preceding illness onset, COVID-19 symptomology, preexisting medical conditions, and patient outcomes, including hospitalization, intensive care unit (ICU) admission, and death. HCP patient health outcomes, overall and stratified by age, were classified as hospitalized, hospitalized with ICU admission, and deaths. The lower bound of these percentages was estimated by including all cases within each age group in the denominators. Upper bounds were estimated by including only those cases with known information on each outcome as denominators. Data reported to CDC are preliminary and can be updated by health departments over time. The upper quartile of the lag between onset date and reporting to CDC was 10 days. Because submitted forms might have missing or unknown information at the time of report, all analyses are descriptive, and no statistical comparisons were performed. Stata (version 15.1; StataCorp) and SAS (version 9.4; SAS Institute) were used to conduct all analyses.

Among 315,531 U.S. COVID-19 cases reported to CDC during February 12–April 9, data on HCP occupational status were available for 49,370 (16%), among whom 9,282 (19%) were identified as HCP (Figure). Data completeness for HCP status varied by reporting jurisdiction; among 12 states that included HCP status on >80% of all reported cases and reported at least one HCP patient, HCP accounted for 11% (1,689 of 15,194) of all reported cases.

**FIGURE:**
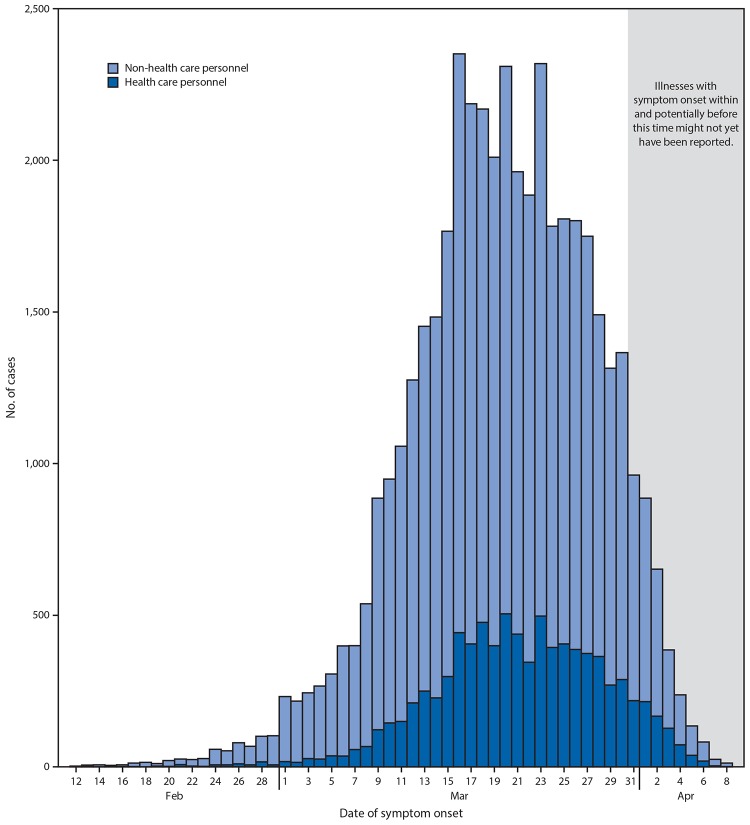
Daily number of COVID-19 cases, by date of symptom onset, among health care personnel and non-health care personnel (N = 43,986)*^,^^†^ — United States, February 12–April 9, 2020 **Abbreviation:** COVID-19 = coronavirus disease 2019. * Onset date was calculated for 5,892 (13%) cases where onset date was missing. This was done by subtracting 4 days (median interval from symptom onset to specimen collection date) from the date of earliest specimen collection. Cases with unknown onset and specimen collection dates were excluded. ^†^ Ten-day window is used to reflect the upper quartile in lag between the date of symptom onset and date reported to CDC.

Among the 8,945 (96%) HCP patients reporting age, the median was 42 years (IQR = 32–54 years); 6,603 (73%) were female (Table 1). Among the 3,801 (41%) HCP patients with available data on race, a total of 2,743 (72%) were white, 801 (21%) were black, 199 (5%) were Asian, and 58 (2%) were other or multiple races. Among 3,624 (39%) with ethnicity specified, 3,252 (90%) were reported as non-Hispanic/Latino and 372 (10%) as Hispanic/Latino. At least one underlying health condition^†^ was reported by 1,779 (38%) HCP patients with available information.

**TABLE 1:** Demographic characteristics, exposures, symptoms, and underlying health conditions among health care personnel with COVID-19 (N = 9,282) — United States, February 12–April 9, 2020

Characteristic (no. with available information)	No. (%)
**Age group (yrs) (8,945)**	
16–44	4,898 (55)
45–54	1,919 (21)
55–64	1,620 (18)
≥65	508 (6)
**Sex (9,067)**	
Female	6,603 (73)
Male	2,464 (27)
**Race (3,801)**	
Asian	199 (5)
Black	801 (21)
White	2,743 (72)
Other*	58 (2)
**Ethnicity (3,624)**	
Hispanic/Latino	372 (10)
Non-Hispanic/Latino	3,252 (90)
**Exposures^†,§^ (1,423)**	
Only health care exposure	780 (55)
Only household exposure	384 (27)
Only community exposure	187(13)
Multiple exposure settings^¶^	72 (5)
Symptoms reported^§,^** (4,707)	
Fever, cough, or shortness of breath^††^	4,336 (92)
Cough	3,694 (78)
Fever^§§^	3,196 (68)
Muscle aches	3,122 (66)
Headache	3,048 (65)
Shortness of breath	1,930 (41)
Sore throat	1,790 (38)
Diarrhea	1,507 (32)
Nausea or vomiting	923 (20)
Loss of smell or taste^¶¶^	750 (16)
Abdominal pain	612 (13)
Runny nose	583 (12)
Any underlying health condition^§,^*** (4,733)	1,779 (38)

**Abbreviation:** COVID-19 = coronavirus disease 2019.

* “Other” includes patients who were identified as American Indian or Alaska Native (16), Native Hawaiian or Other Pacific Islander (22), or two or more races (20).

^†^ Cases were included in the denominator if the patient reported a known contact with a laboratory-confirmed COVID-19 patient within the 14 days before illness onset in a health care, household, or community setting.

^§^ Responses include data from standardized fields supplemented with data from free-text fields.

^¶^ Includes all patients with contact reported in more than one of these settings: health care, household, and community.

** Cases were included in the denominator if the patient had a known symptom status for fever, cough, shortness of breath, nausea or vomiting, and diarrhea. HCP with mild or asymptomatic infections might have been less likely to be tested, thus less likely to be reported.

^††^ Includes all patients with at least one of these symptoms.

^§§^ Patients were included if they had information for either measured or subjective fever variables and were considered to have a fever if “yes” was indicated for either variable.

^¶¶^ Symptom data on loss of smell or taste was extracted only from free-text symptom fields, thus the proportion with this symptom is likely an underestimate.

*** Preexisting medical conditions and other risk factors (yes, no, or unknown) included the following: chronic lung disease (inclusive of asthma, chronic obstructive pulmonary disease, and emphysema); diabetes mellitus; cardiovascular disease; chronic renal disease; chronic liver disease; immunocompromised condition; neurologic disorder, neurodevelopmental or intellectual disability; pregnancy; current smoking status; former smoking status; or other chronic disease.

Among 1,423 HCP patients who reported contact with a laboratory-confirmed COVID-19 patient in either health care, household, or community settings, 780 (55%) reported having such contact only in a health care setting within the 14 days before their illness onset; 384 (27%) reported contact only in a household setting; 187 (13%) reported contact only in a community setting; 72 (5%) reported contact in more than one of these settings. Among HCP patients with data available on a core set of signs and symptoms,^§^ a total of 4,336 (92%) reported having at least one of fever, cough, shortness of breath. Two thirds (3,122, 66%) reported muscle aches, and 3,048 (65%) reported headache. Loss of smell or taste was written in for 750 (16%) HCP patients as an “other” symptom.

Among HCP patients with data available on age and health outcomes, 6,760 (90%) were not hospitalized, 723 (8%–10%) were hospitalized, 184 (2%–5%) were admitted to an ICU, and 27 (0.3%–0.6%) died (Table 2). Although only 6% of HCP patients were aged ≥65 years, 10 (37%) deaths occurred among persons in this age group.

**TABLE 2:** Hospitalizations,* intensive care unit (ICU) admissions,^†^ and deaths,^§^ by age group among health care personnel with COVID-19 — United States, February 12–April 9, 2020

Age group^¶^ (yrs) (no. of cases)	Outcome, no. (%)**		
	Hospitalization^††^	ICU admission	Death
16–44 (4,898)	260 (5.3–6.4)	44 (0.9–2.2)	6 (0.1–0.3)
45–54 (1,919)	178 (9.3–11.1)	51 (2.7–6.3)	3 (0.2–0.3)
55–64 (1,620)	188 (11.6–13.8)	54 (3.3–7.5)	8 (0.5–1.0)
≥65 (508)	97 (19.1–22.3)	35 (6.9–16.0)	10 (2.0–4.2)
**Total (8,945)**	**723 (8.1–9.7)**	**184 (2.1–4.9)**	**27 (0.3–0.6)**

**Abbreviation:** COVID-19 = coronavirus disease 2019.

* Hospitalization status known for 7,483 (84%) patients.

^†^ ICU status known for 3,739 (42%) patients.

^§^ Death outcomes known for 4,407 (49%) patients.

^¶^ Age status known for 8,945 (96%) patients.

** Lower bound of range = number of persons hospitalized, admitted to ICU, or who died among total in age group; upper bound of range = number of persons hospitalized, admitted to ICU, or who died among total in age group with known hospitalization status, ICU admission status, or death.

^††^ Hospitalization status includes hospitalization with or without ICU admission.

## Discussion

As of April 9, 2020, a total of 9,282 U.S. HCP with confirmed COVID-19 had been reported to CDC. This is likely an underestimation because HCP status was available for only 16% of reported cases nationwide. HCP with mild or asymptomatic infections might also have been less likely to be tested, thus less likely to be reported. Overall, only 3% (9,282 of 315,531) of reported cases were among HCP; however, among states with more complete reporting of HCP status, HCP accounted for 11% (1,689 of 15,194) of reported cases. The total number of COVID-19 cases among HCP is expected to rise as more U.S. communities experience widespread transmission. Compared with reports of COVID-19 patients in the overall populations of China and Italy (*4*,*5*), reports of HCP patients in the United States during February 12–April 9 were slightly younger, and a higher proportion were women; this likely reflects the age and sex distributions among the U.S. HCP workforce. Race and ethnicity distributions among HCP patients reported to CDC are different from those in the overall U.S. population but are more similar to those in the HCP workforce.^¶^^,^**

Among HCP patients who reported having contact with a laboratory-confirmed COVID-19 patient in health care, household, or community settings, the majority reported contact that occurred in health care settings. However, there were also known exposures in households and in the community, highlighting the potential for exposure in multiple settings, especially as community transmission increases. Further, transmission might come from unrecognized sources, including presymptomatic or asymptomatic persons (*6*,*7*). Together, these exposure possibilities underscore several important considerations for prevention. Done alone, contact tracing after recognized occupational exposures likely will fail to identify many HCP at risk for developing COVID-19. Additional measures that will likely reduce the risk for infected HCP transmitting the virus to colleagues and patients include screening all HCP for fever and respiratory symptoms at the beginning of their shifts, prioritizing HCP for testing, and ensuring options to discourage working while ill (e.g., flexible and nonpunitive medical leave policies). Given the evidence for presymptomatic and asymptomatic transmission (*7*), covering the nose and mouth (i.e., source control) is recommended in community settings where other social distancing measures are difficult to maintain.^††^ Assuring source control among all HCP, patients, and visitors in health care settings is another promising strategy for further reducing transmission. Even if everyone in a health care setting is covering their nose and mouth to contain their respiratory secretions, it is still critical that, when caring for patients, HCP continue to wear recommended personal protective equipment (PPE) (e.g., gown, N95 respirator [or facemask if N95 is not available], eye protection, and gloves for COVID-19 patient care). Training of HCP on preventive measures, including hand hygiene and PPE use, is another important safeguard against transmission in health care settings.

Among HCP with COVID-19 whose age status was known, 8%–10% were reported to be hospitalized. This is lower than the 21%–31% of U.S. COVID-19 cases with known hospitalization status described in a recent report (*8*) and might reflect the younger median age (42 years) of HCP patients compared with that of reported COVID-19 patients overall, as well as prioritization of HCP for testing, which might identify less severe illness. Similar to earlier findings (*8*), increasing age was associated with a higher prevalence of severe outcomes, although severe outcomes, including death, were observed in all age groups. Preliminary estimates of the prevalence of underlying health conditions among all patients with COVID-19 reported to CDC through March 2020 (*9*) suggested that 38% had at least one underlying condition, the same percentage found in this HCP patient population. Older HCP or those with underlying health conditions (*8*,*9*) should consider consulting with their health care provider and employee health program to better understand and manage their risks regarding COVID-19. The increased prevalence of severe outcomes in older HCP should be considered when mobilizing retired HCP to increase surge capacity, especially in the face of limited PPE availability^§§^; one consideration is preferential assignment of retired HCP to lower-risk settings (e.g., telemedicine, administrative assignments, or clinics for non–COVID-19 patients).

The findings in this report are subject to at least five limitations. First, approximately 84% of patients were missing data on HCP status. Thus, the number of cases in HCP reported here must be considered a lower bound because additional cases likely have gone unidentified or unreported. Second, among cases reported in HCP, the amount of missing data varied across demographic groups, exposures, symptoms, underlying conditions, and health outcomes; cases with available information might differ systematically from those without available information. Therefore, additional data are needed to confirm findings about the impact of potentially important factors (e.g., disparities in race and ethnicity or underlying health conditions among HCP). Third, additional time will be necessary for full ascertainment of outcomes, such as hospitalization status or death. Fourth, details of occupation and health care setting were not routinely collected through case-based surveillance and, therefore, were unavailable for this analysis. Finally, among HCP patients who reported contact with a confirmed COVID-19 patient in a health care setting, the nature of this contact, including whether it was with a patient, visitor, or other HCP, and the details of potential occupational exposures, including whether HCP were unprotected (i.e., without recommended PPE) or were present during high risk procedures (e.g., aerosol-generating procedures) are unknown (*10*).

It is critical to make every effort to ensure the health and safety of this essential national workforce of approximately 18 million HCP, both at work and in the community. Surveillance is necessary for monitoring the impact of COVID-19-associated illness and better informing the implementation of infection prevention and control measures. Improving surveillance through routine reporting of occupation and industry not only benefits HCP, but all workers during the COVID-19 pandemic.

**Summary**

**What is already known about this topic?**

Limited information is available about COVID-19 infections among U.S. health care personnel (HCP).

**What is added by this report?**

Of 9,282 U.S. COVID-19 cases reported among HCP, median age was 42 years, and 73% were female, reflecting these distributions among the HCP workforce. HCP patients reported contact with COVID-19 patients in health care, household, and community settings. Most HCP patients were not hospitalized; however, severe outcomes, including death, were reported among all age groups.

**What are the implications for public health practice?**

It is critical to ensure the health and safety of HCP, both at work and in the community. Improving surveillance through routine reporting of occupation and industry not only benefits HCP, but all workers during the COVID-19 pandemic.

## Footnotes

* https://www.cdc.gov/coronavirus/2019-ncov/php/reporting-pui.html.

† Preexisting medical conditions and other risk factors (yes, no, or unknown) included the following: chronic lung disease (inclusive of asthma, chronic obstructive pulmonary disease, and emphysema); diabetes mellitus; cardiovascular disease; chronic renal disease; chronic liver disease; immunocompromised condition; neurologic disorder, neurodevelopmental or intellectual disability; pregnancy; current smoker; former smoker; or other chronic disease. Data available for 4,733 (51%) HCP.

§ Cases were included in the denominator if the patient had a known symptom status for fever, cough, shortness of breath, nausea or vomiting, and diarrhea. Data available for 4,707 (51%) HCP.

¶ https://www.bls.gov/cps/tables.htm#charemp.

** https://data.census.gov/cedsci/.

†† https://www.cdc.gov/coronavirus/2019-ncov/prevent-getting-sick/cloth-face-cover.html.

§§ https://www.cdc.gov/coronavirus/2019-ncov/hcp/ppe-strategy/index.html.
